# An in-house real-time polymerase chain reaction: standardisation and comparison with the Cobas Amplicor HBV monitor and Cobas AmpliPrep/Cobas TaqMan HBV tests for the quantification of hepatitis B virus DNA

**DOI:** 10.1590/0074-02760150415

**Published:** 2016-02

**Authors:** Ana Paula de Torres Santos, José Eduardo Levi, Marcilio Figueiredo Lemos, Samira Julien Calux, Isabel Takano Oba, Regina Célia Moreira

**Affiliations:** 1 Universidade de São Paulo, Faculdade de Medicina, Hospital das Clínicas, Divisão de Laboratório Central, Laboratório de Imunologia, São Paulo, SP, Brasil; 2 Universidade de São Paulo, Instituto de Medicina Tropical, Laboratório de Virologia, São Paulo, SP, Brasil; 3 Instituto Adolfo Lutz, Centro de Virologia, Núcleo de Doenças Sanguíneas e Sexuais, São Paulo, SP, Brasil

**Keywords:** HBV viral load, Cobas Amplicor HBV monitor, Cobas TaqMan HBV, HBVDNA, in-house rtPCR

## Abstract

This study aimed to standardise an in-house real-time polymerase chain reaction (rtPCR) to allow quantification of hepatitis B virus (HBV) DNA in serum or plasma samples, and to compare this method with two commercial assays, the Cobas Amplicor HBV monitor and the Cobas AmpliPrep/Cobas TaqMan HBV test. Samples from 397 patients from the state of São Paulo were analysed by all three methods. Fifty-two samples were from patients who were human immunodeficiency virus and hepatitis C virus positive, but HBV negative. Genotypes were characterised, and the viral load was measure in each sample. The in-house rtPCR showed an excellent success rate compared with commercial tests; inter-assay and intra-assay coefficients correlated with commercial tests (r = 0.96 and r = 0.913, p < 0.001) and the in-house test showed no genotype-dependent differences in detection and quantification rates. The in-house assay tested in this study could be used for screening and quantifying HBV DNA in order to monitor patients during therapy.

Hepatitis B virus (HBV) infection is a major health problem and causes significant levels of morbidity and mortality worldwide. Approximately 30% of the world’s population, or about two billion people, have serological evidence of past or current HBV infection. Out of these, it is estimated that approximately 360 million have chronic infection and 600,000 die annually due to acute hepatitis, cirrhosis, and hepatocellular carcinoma (Valla 2003). According to the Population-Based Prevalence Study of viral hepatitis including all major cities in Brazil, the prevalence of the main biomarker indicating exposure to HBV (hepatitis B core antigen) was 7.4% (MS 2010).

A combination of biochemical and serological markers in addition to histological features are used as the main tools for the diagnosis of HBV infection. The interpretation of transaminase analysis results and research into antigens and/or antibodies allow the sorting and tracking of HBV infection cases.

While screening for viral DNA is not recommended for the diagnosis of HBV infection, the quantification of HBV DNA is crucial for evaluating responses to treatment and tracking the emergence of mutant viral strains. HBV DNA screening must be used in situations where there are signs of hepatocellular damage, which often indicates the development of mutant strains during the course of antiviral therapy. In these circumstances, the serological markers of viral replication could appear negative, calling for further biomolecular testing. High concentrations of viral DNA can be detected in serum, indicating active viral replication (Welzel et al. 2006, Galli et al. 2008).

In order to establish criteria for diagnosis and treatment and to organise the use of molecular tests for HBV in the public health service, the Brazilian Ministry of Health updated the clinical protocol and therapeutic guidelines for HBV infection in 2011 (funed.mg.gov.br/wp-content/uploads/2011/07/prot_clinico_diretrizes_terapeuticas_hep_B.pdf). In almost all situations, it is necessary to monitor the patient and employ methods of determining the viral load (VL). The Brazilian Ministry of Health has collaborated with public health laboratories, and it is important that these institutions can meet diagnostic demand by developing standardised and economically viable alternatives to commercial tests.

Quantitative HBV DNA analysis can be carried out using a number of different methods: signal amplification (bDNA), polymerase chain reaction (PCR), and real-time PCR (rtPCR) (TaqMan PCR) (Lu et al. 2006). The TaqMan rtPCR method is a modification of the traditional PCR procedure, which identifies the target with greater sensitivity and specificity with the added benefit of short reaction times. This procedure relies on the detection and quantification of a fluorescent signal, whose intensity is directly proportional to the amount of amplified product created during the reaction (Zhao et al. 2005, Lu et al. 2006). The objective of this study was to standardise the in-house rtPCR method used in a public health laboratory - the Laboratory of Viral Hepatitis, Adolfo Lutz Institute, state of São Paulo (SP), Brazil - and to compare the results obtained to those gathered using commercially available kits, namely Cobas Amplicor HBV monitor (CAHM) and Cobas AmpliPrep/Cobas TaqMan (CAP/CTM).

## SUBJECTS, MATERIALS AND METHODS

*Patients and samples* - This study included 397 samples of serum or plasma from patients with chronic HBV infection who were starting or following-up public health service treatment in SP. The samples were received, divided into aliquots, numbered sequentially, and stored at -20ºC in the Laboratory of Viral Hepatitis, Adolfo Lutz Institute, from May 2009-June 2011. From these 397 samples, 52 serum samples from individuals who were HBV seronegative, but human immunodeficiency virus (HIV) seropositive (27 samples) or hepatitis C virus positive (25 samples), were selected only for specificity tests. This study was approved by the Ethical Committee of the Adolfo Lutz Institute (protocol 1872/2009).

*In-house rtPCR HBV DNA assay* - To standardise the in-house rtPCR procedure, we employed a serum sample from the World Health Organization containing well a characterised (10^5^ IU/mL) level of DNA. This standard was used to construct a standard curve, ranging from an undetected VL to 100,000 IU/mL of HBV DNA.

*Primers and probes standardise* - The primers and probes used to standardise the in-house quantitative rtPCR were specific to the virus “core” and “S” regions of the HBV genome. For testing purposes, 10 serum samples with known VLs and genotypes were used. VLs ranged from 70-25.200 UI/mL and genotypes were A, D, and C; three sets of primers and probes were used. Initially, the primers and probes were resuspended at a concentration of 100 mM and subsequently a stock was prepared from concentrated stock (10X). Just before performing the reactions, a second stock was prepared in concentrations suitable for each reaction.

Table I shows the primer and probe sequences, their concentrations, and the authors that described them.

**TABLE I:** Primers and probes sequences, concentration, and regions

Primer/probe	Region	Concentration (µM)	References
5’CAACCTCCAATCACTCACCAA 3’	S	Primers: 0.9 Probe: 0.2	Compston et al. (2008)
3’ATATGATAAAACGCCGCAGACAC 5’			
5’FAM CTCCTCCAATTTGTCCTGGTTATCGCT 3’ BHQ1			
5’CAACCTCTTGTCCTCCAACTTGT 3’	S	Primers: 0.6 Probe: 0.4	Drosten et al. (2000)
3’AACCTCCTGTCCTCCAACTTGT 5’			
5’CAACCTGTTGTCCTCCAATTTGT 3’			
3’GATGAGGCATAGCAGCAGGAT 5’			
5’FAM-ATCGCTGGATGTGTCTGCGGCGTT-BHQ1 3’			
3’GCCCCTATCTTATCAACACTTCCGGAAAC5’	Core	Primer: 0.3 Probe: 0.1	Aytay et al. (2004)
5’FAM-TGTTGTTAGACGACGAGGCAGGTCCCTAG-BHQ1 3’			
3’GATACTAACATTGAGATTCCCGAGATTG5’			

To perform rtPCR, HBV DNA was extracted from 200 μL of serum or plasma using a QIAamp DNA Mini commercial kit (QIAGEN, Germany), following the manufacturer’s instructions. The amplification reaction was performed on an Applied Biosystems 7300 Real-Time PCR System (Applied Biosystems, USA) with cycling conditions of 50ºC for 2 min, 95ºC for 10 min, 40 cycles of 95ºC for 15 s, followed by 60ºC for 1 min. Samples were considered positive when the exponential amplification curve crossed the predetermined threshold and the profiles of signals emitted by the fluorescent reporter and the passive reference dye were in agreement with predetermined parameters. The results were analysed using ABI Prism software (Applied Biosystems).

*CAHM assay* - This commercial test was performed following the manufacturer’s instructions. CAHM is based on four main processes: sample preparation, amplification by PCR using specific primers complementary to HBV, hybridisation of the amplified products with specific probes, and the detection of amplified products for colorimetric determination. The test simultaneously allows PCR amplification and quantitation of HBV DNA and an internal quantitation standard. The measurement range of the CAHM is 60-38,000 IU/mL.

*CAP/CTM assay* - This test allows the automated preparation of samples followed by automated amplification and detection of target HBV DNA and an internal quantitation standard. The reagent master mix contains primer pairs and probes specific for HBV DNA and the internal quantitation standard. Detection of amplified DNA is conducted by means of a double-labelled oligonucleotide probe specific for the target and the internal quantitation standard, which allows independent amplicon identification of both targets. The measurement range of the CAP/CTM assay is 20-170,000,000 IU/mL.

*Statistical analysis* - Statistical analysis was performed using Statistical Package for the Social Sciences for Windows v.13 and EP Evaluator v.9.3.0.448. Quantitative correlation results from two assays were determined by a scatter graph and Pearson’s coefficient analysis after the data was transformed logarithmically. Kappa (K) indices and receiving operating characteristics (ROC) curves were also calculated. The reproducibility of the in-house rtPCR protocol was assessed by testing 26 samples with different VLs in triplicate on three different days.

## RESULTS

*Samples* - Patient age ranged from one-82 years further, 178 (51.6%) samples were from male patients and 167 (48.4%) were from female patients.

A total of 345 samples of serum and/or plasma were analysed in this study. In total, 142 samples were positive according to CAHM, 94 samples were positive according to CAP/CTM, and 109 samples were negative according to both tests.

*Primers and probes* - After testing, the primers and probes described by Drosten et al. (2000) provided the best results, detecting samples with a variety of different VLs and HBV genotypes. This set was employed in all in-house rtPCR reactions.

*Genotypes* - From 236 samples with VLs detectable by CAHM and/or CAP/CTM, it was possible to characterise the genotype in 217 (92%). The genotypes were genotype A (104 samples), genotype D (90 samples), genotype F (12 samples), genotype C (10 samples), and genotype G (1 sample).

**Fig. 1:**
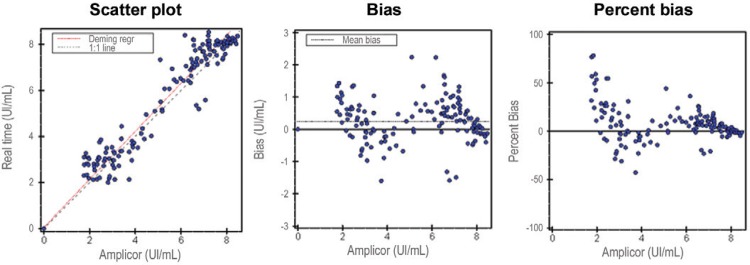
: validity of the real-time polymerase chain reaction using Cobas Amplicor HBV monitor as the gold standard.

*Validity* - The HBV VL quantified by in-house rtPCR was compared with the results of commercial tests. CAHM showed strong a correlation to the in-house PCR (r = 0.977, p < 0.001, k = 100%) and errors were distributed around zero. The regression line (red, in Fig. 1) approximately superimposed the line (dashed black, in Fig. 1) corresponding to perfect agreement, also suggesting excellent agreement. The regression coefficient was close to 1 [1.018 (0.983-1.052)] (Fig. 1).

**Fig. 2:**
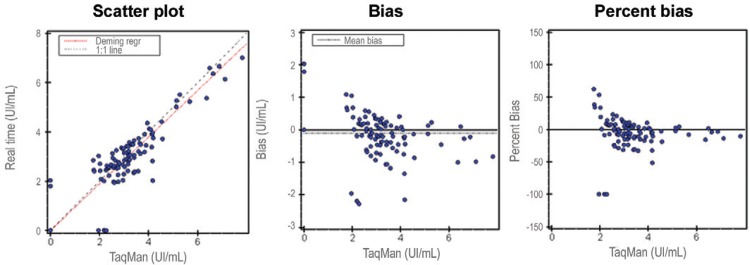
: validity of the real-time polymerase chain reaction using Cobas AmpliPrep/Cobas TaqMan as the gold standard.

Similar results were observed when the in-house rtPCR assay was compared to CAP/CTM. There were significant correlations between the measurements (r = 0.96, p < 0.001, k = 94.4%) and the regression coefficient was close to 1 (0.913) (Fig. 2).

**Fig. 3:**
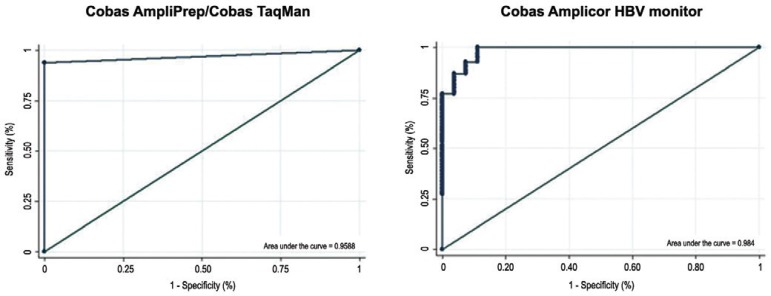
: sensitivity and specificity of in-house real-time polymerase chain reaction compared to Cobas Amplicor HBV monitor and Cobas AmpliPrep/Cobas TaqMan (analysis of receiver operator characteristics curves).

Sensitivity and specificity of in-house rtPCR compared to both commercial tests show an excellent performance. These data are shown in Fig. 3.

Table II shows a summary of the accuracy of rtPCR comparing to commercial kits.

**TABLE II:** Accuracy of real-time polymerase chain reaction (rtPCR) comparing to commercial kits Cobas Amplicor HBV monitor (CAHM) and Cobas AmpliPrep/Cobas TaqMan (CAP/CTM)

In-house rtPCR	CAHM	CAP/CTM
Pearson’s correlation coefficient	0.977 (p < 0.001)	0.960 (p < 0.001)
Coefficient’s regression (Slope)	1.018 (0.983-1.052)	0.913 (0.874-0.953)
Agreement (%)	100 (97.7-100)	97.2 (93.6-98.8)
Kappa index (%)	100 (100-100)	94.4 (89.6-99.2)
Sensitivity (%)	100 (100-100)	96.8 (93.2-100)
Specificity (%)	100 (100-100)	97.7 (94.4-100)
Positive predictive value (%)	100 (100-100)	97.8 (94.9-100)
Negative predictive value (%)	100 (100-100)	96.5 (92.6-100)

*Reproducibility* - Tables IIIand IV show the reproducibility results of inter-assay analysis after testing 26 serum/plasma samples tested in triplicates and on different days.

**TABLE III:** Reproducibility results of inter-assay analysis after testing 26 serum/plasma samples tested in triplicates and on different days

Sample number	Mean viral load (Log_10_)			SD	VC (%)

	Run 1	Run 2	Run 3		
15	2.95	2.80	2.51	0.224804	8.17
24	8.85	9.13	9.05	0.139997	1.55
29	8.29	8.46	8.41	0.089059	1.06
36	3.45	3.22	3.01	0.222690	6.90
41	3.03	3.49	3.34	0.235490	7.17
47	4.12	4.01	3.85	0.136158	3.41
49	3.30	3.31	3.17	0.076007	2.33
52	8.42	8.65	8.57	0.112566	1.32
60	6.82	6.91	6.74	0.084088	1.23
67	8.38	8.59	8.53	0.106412	1.25
70	8.65	8.86	8.78	0.106277	1.21
73	3.61	3.73	3.47	0.133094	3.69
76	4.34	4.23	4.12	0.110777	2.62
80	3.91	3.96	3.63	0.179675	4.69
85	3.11	2.98	2.88	0.112976	3.78
91	3.40	2.90	2.95	0.277211	8.98
97	8.74	8.88	8.93	0.096687	1.09
109	4.37	4.27	4.21	0.082620	1.93
110	4.45	4.49	4.30	0.098887	2.24
115	3.09	2.82	2.66	0.220767	7.72
121	3.13	3.11	3.08	0.027903	0.90
123	8.66	8.89	8.86	0.124552	1.41
125	8.03	8.20	8.17	0.093246	1.15
130	3.39	3.35	3.28	0.053025	1.59
134	8.74	9.02	8.94	0.144338	1.62
138	5.26	3.17	3.34	1.159088	29.53

SD: standard deviation; VC: variation coefficient.

**TABLE IV:** Results of reproducibility analysis of the in-house real-time polymerase chain reaction method using the Pearson’s correlation coefficient (r) and intra-assay coefficient (ICC)

Replicates	Measurement 2	Measurement 3
Measurement 1	r = 0.985 (0.000) ICC = 0.983 (0.97 -0.996)	r = 0.989 (0.000) ICC = 0.984 (0.972-0.996)
Measurement 2	-	r = 0.999 (0.000) ICC = 0.998 (0.997-0.999)

*Specificity* - DNA was extracted from the samples that showed negative results to HBV and was processed for rtPCR. All samples yielded negative results, demonstrating 100% specificity. Samples showed no cross-reactivity against HBV.

## DISCUSSION

In the present study, the serum or plasma samples were randomly chosen and the group was composed of both men and women. Although the literature suggests that HBV affects men more than women (MS 2011), there were no statistically significant differences in relation to gender in our study.

There are many commercially available assays that are routinely used to quantify HBV DNA in diagnostic laboratories. Most commercial quantitative assays have an upper detection limit and clinical samples therefore need to be diluted and retested to determine VLs accurately. The in-house rtPCR method tested in this study has an upper detection limit of approximately 10^8^ IU/mL. Therefore, samples do not require dilution and do not need to be retested. The detection of a VL of approximately 10^8^ IU/mL has also been reported in other studies (Daniel et al. 2009).

Initially, we identified primers and probes in the literature that had demonstrated good performance in standardised tests. We selected three pairs of primers and tested them in the same conditions and on the same samples. The samples were characterised according to VL, as it was necessary to use reagents that would allow the analysis of samples independent of VL and also to be able to quantify samples of all genotypes. We chose the primer set designed by Drosten et al. (2000) because of these primers allowed the detection of the different genotypes and VLs. Drosten et al. (2000) demonstrated the effectiveness of these primers and our results correlate with these findings.

Results of the analysis of 345 clinical samples obtained using the in-house rtPCR, CAHM, and CAP/CTM were concordant. k values were 100% and 94.4%, and sensitivity and specificity indices were close to 100%. When comparing HBV DNA levels in 246 serum samples, the results were very strongly correlated (r = 0.977 and 0.944).

Some authors have standardised in-house rtPCR testes for quantification and genotypes HBV. Santos et al. (2014) developed an in-house rtPCR ultra sensitive and effective as a new nucleic acid amplification techniques alternative and compared VL values to Acrometrix^®^ HBV DNA kit. They found strong correlation (r^2^ = 0.998, p < 0.0001) between the tests and an efficiency of 94.06%.

Becker et al. (2013) standardised an in-house rtPCR for genotyping different strains of HBV. These authors conducted a study employing set of primers specifically for genotype A, D, and F because of these are more common in our country. These authors observed good performance in identify the genotypes, but this test failed to quantify HBV VL in 29.6% of the positive samples.

Comparing to another Brazilian study, it´s possible observed the excellent performance of our test and that it could be an alternative to HBV detection and quantification in serum or plasma.

EP Evaluator revealed a strong correlation between the different systems and errors were distributed around zero, demonstrating excellent concordance between the in-house PCR and the CAHM. Excellent agreement was also demonstrated by approximate superimposition of the regression line and the line corresponding to perfect agreement. These results corresponded to a regression coefficient of close to 1.Shi et al. (2008) conducted a similar study comparing the Fosun Real-Time PCR HBV assay with CAP/CTM. These authors found a strong correlation between CAP/CTM and the Fosun Real-Time PCR assay. The study was conducted in China and, when assessing 118 samples, a correlation coefficient of 0.948 and a p-value of < 0.001 were determined. Similar results were observed when we compared our in-house rtPCR with CAP/CTM, with significant correlations and regression coefficients close to 1. In most cases, the results were concordant.

Sensitivity and specificity values were above 96%, demonstrating excellent PCR accuracy in both comparisons. The sensitivity and specificity indices were excellent and ROC curves demonstrated results close to 1. There were no differences dependent on the use of serum or plasma.

Commercial tests commonly use a sample volume of 1,000 µL, while our in-house rtPCR assay only used 200 µL. This can minimise the risks associated with collecting the sample for the patient and reduce the number samples rejected due to insufficient volume.

The in-house rtPCR method developed in this study has an added value in reducing the economic burden of analysis, while simultaneously reducing the running time of the test compared to commercial kits (mean time to obtain results, including extraction = 4 h).

The ROC curve is the most efficient way to demonstrate the relationship between sensitivity and specificity between two tests, with this relationship usually being antagonistic. It is an important tool for measuring and specifying the performance issues of diagnostic tests. Analysis of ROC curves evaluating the in-house rtPCR method compared to CAHM and CAP/CTM confirmed the accuracy of the in-house method. The area under the curve when comparing the in-house rtPCR to CAHM was close to 0.984 and when comparing to CAP/CTM was about 0.9688. These results were very close to 1, which would indicate 100% sensitivity and 100% specificity.

As expected, the most prevalent genotypes in the population were A and D and these results are consistent with other studies on the prevalence of genotypes in Brazil (Bottecchia et al. 2008, Alvarado-Mora & Pinho 2013). In this study, we also identified one strain of the G genotype, which is rare in our population. The sample in which genotype G was identified was from patient with HIV. This matches the result reported by da Silva et al. (2010). Genotypes A and D are the most prevalent worldwide (including Brazil), while genotype F is the prevalent genotype in the Americas and genotype C is characteristic of Asian populations and also quite common in the city of São Paulo. Compri et al. (2012) identified genotypes A, D, and C in children, adolescents, and their families in SP. Moraes et al. (1996) found genotypes A, D, and F in the state of Rio de Janeiro. These same genotypes were also described in the populations of the city of Goiânia, state of Goiás, and of the state of Santa Catarina (Teles et al. 1999, Carrilho et al. 2004, Viana et al. 2005, Alcalde et al. 2009, Becker et al. 2013).

However, the main objective in identifying HBV genotypes in this study, besides characterising the population, was to verify that our test was able to detect all the genotypes present in serum samples with the same efficacy as commercially available kits, although we could evaluate only 10 samples from genotype C, 12 samples from genotype F, and only one from genotype G. Our results were satisfactory because in addition to detecting the different viral strains, the VLs measured were the same when employing different methods, i.e., in-house or commercial. Furthermore, the sample with the rare G genotype was efficiently detected and quantified by our method. These results demonstrate that the excellent performance of our in-house test was independent of genotype and VL.

The in-house rtPCR had good reproducibility with low average variability between replicates. Reproducibility analysis was important for assessing the validity of the assay and for verifying whether variation occurs depending on the day of analysis. The variability between replicates appears to be very small and the differences between measurements were mostly zero, with the occurrence of only one anomaly. Further studies are necessary to know the performance of the in-house rtPCR in another laboratories, especially in regions where human resources or financial founds are scarce.

Thus, our results showed a high correlation between our in-house rtPCR and two commercially available kits. The test showed high reproducibility with low variability between results obtained in various tests. The test was able to detect different genotypes and VLs.

Considering the analytical sensitivity, accuracy, specificity, and wide range of quantitative detection offered by the in-house rtPCR developed in this study, we conclude that the test represents an excellent alternative to commercially available kits. This test can be used for monitoring patients with chronic HBV infection and can be immediately deployed in diagnostic or clinical research laboratories.

## References

[B1] Alcalde R, Melo FL, Nishiya A, Ferreira SC, Langhi MD, Fernandes SS, Marcondes LA, Duarte AJS, Casseb J (2009). Distribution of hepatitis B virus genotypes and viral
load levels in Brazilian chronically infected patients in São Paulo
city. Rev Inst Med Trop Sao Paulo.

[B2] Alvarado-Mora MV, Pinho JRR (2013). Distribuition of HBV genotypes in Latin
America. Antivir Ther.

[B3] Aytay S, Ohagen A, Busch MP, Alford B, Chapman JR, Lazo A (2004). Development of a sensitive PCR inhibition method to
demonstrate HBV nucleic acid inactivation. Transfusion.

[B4] Becker CE, Kretzmann NA, Mattos AA, Veiga ABG (2013). Melting curve analysis for the screening of hepatitis B
virus genotypes A, D, and F in patients from a general hospital in southern
Brazil. Arq Gastroenterol.

[B5] Bottecchia M, Souto FJD, Kycia MRO, Amendola M, Brandão CE, Niel C, Gomes SA (2008). Hepatitis B virus genotypes and resistance mutations in
patients under long term lamivudine therapy: characterization of genotype G in
Brazil. BMC Microbiol.

[B6] Carrilho FJ, Moraes CR, Pinho JRR, Mello IM, Bertolini DA, Lemos MF, Moreira RC, Bassit LC, Cardoso RA, Ribeiro-dos-Santos G, Silva LC (2004). Hepatitis B virus infection in haemodialysis centres
from Santa Catarina state, southern Brazil. Predictive risk factors for infection
and molecular epidemiology. BMC Public Health.

[B7] Compri AP, Miura I, Porta G, Lemos MF, Saraceni CP, Moreira RC (2012). Hepatitis B virus infection in children, adolescents,
and their relatives: genotype distribution and precore and core gene
mutations. Rev Soc Bras Med Trop.

[B8] Compston LI, Sarkobie F, Li C, Candotti D, Opare-Sem O, Allain JP (2008). Multiplex real-time PCR for the detection and
quantification of latent and persistent viral genomes in cellular or plasma blood
fractions. J Virol Methods.

[B9] Silva AC, Spina AMM, Lemos MF, Oba IT, Guastini CF, Gomes-Gouvêa MS, Pinho JRR, Mendes-Correa MCJ (2010). Hepatitis B genotype G and high frequency of lamivudine
- resistance mutations among human immunodeficiency virus/hepatitis B virus
co-infected patients in Brazil. Mem Inst Oswaldo Cruz.

[B10] Daniel HDJ, Fletcher JG, Chandy GM, Abraham P (2009). Quantitation of hepatitis B virus DNA in plasma using a
sensitive cost-effective “in-house” real-time PCR assay. Indian J Med Microbiol.

[B11] Drosten C, Weber M, Seifried E, Roth WK (2000). Evaluation of a new PCR assay with competitive internal
control sequence for blood donor screening. Transfusion.

[B12] Galli C, Orlandini E, Penzo L, Badiale R, Caltran G, Valverde S, Gessoni G (2008). What is the role of serology for the study of chronic
hepatitis B virus infection in the age of molecular biology?. J Med Virol.

[B13] Lu YQ, Han JX, Qi P, Xu W, Zu YH, Zhu B (2006). Rapid quantification of hepatitis B virus DNA by
real-time PCR using efficient TaqMan probe and extraction of virus
DNA. World J Gastroenterol.

[B14] Moraes MTB, Gomes SA, Niel C (1996). Sequence analysis of pre-S/S gene of hepatitis B virus
strains of genotypes A, D, and F isolated in Brazil. Arch Virol.

[B15] MS - Ministério da Saúde Brasil (2010). Estudo de prevalência de base populacional das infecções pelos
vírus das hepatites A, B e C nas capitais do Brasil.

[B16] MS - Ministério da Saúde Brasil (2011). Protocolo clínico e diretrizes terapêuticas para o tratamento
da hepatite viral crônica B e coinfecções.

[B17] Santos AO, Souza LFB, Borzacov LM, Villalobos-Salcedo JM, Vieira DS (2014). Development of cost-effective real-time PCR test: to
detect a wide range of HBV DNA concentrations in the western Amazon Region of
Brazil. Virol J.

[B18] Shi M, Zhang Y, Zhu YH, Zhang J, Xu WJ (2008). Comparison of real-time polymerase chain reaction with
the COBAS Amplicor test for quantitation of hepatitis B virus DNA in serum
samples. World J Gastroenterol.

[B19] Teles SA, Martins RMB, Vanderborght B, Stuyver L, Gaspar AMC, Yoshida CFT (1999). Hepatitis B virus: genotypes and subtypes in Brazilian
hemodialysis patients. Artif Organs.

[B20] Valla DC (2003). EASL International Consensus Conference on Hepatitis B
(short version). J Hepatol.

[B21] Viana S, Paraná R, Moreira RC, Compri AP, Macedo V (2005). High prevalence of hepatitis B virus and hepatitis D
virus in the western Brazilian Amazon. Am J Trop Med Hyg.

[B22] Welzel TM, Miley WJ, Parks TL, Goedert JJ, Whitby D, Ortiz-Conde BA (2006). Real-time PCR assay for detection and quantification of
hepatitis B virus genotypes A to G. J Clin Microbiol.

[B23] Zhao JR, Bai YJ, Zhang QH, Wan Y, Li D, Yan XJ (2005). Detection of hepatitis B virus DNA by real-time PCR
using TaqMan-MGB probe technology. World J Gastroenterol.

